# Use of LigaSure vessel sealing system versus conventional axillary dissection in breast cancer patients: a retrospective comparative study

**DOI:** 10.1186/s12893-022-01888-2

**Published:** 2022-12-22

**Authors:** V. Wienerroither, R. Hammer, P. Kornprat, H. Schrem, D. Wagner, H. J. Mischinger, A. El-Shabrawi

**Affiliations:** 1 Department of General, Visceral and Transplant Surgery, Medical University of Graz, Auenbruggerplatz 29, 8036 Graz, Austria; 2 Department of Surgery, LKH Graz II, Graz, Austria

**Keywords:** Axillary lymph node dissection, Breast cancer, Thermal sealing system, Hemostasis, LigaSure™, Electrocautery

## Abstract

**Background:**

In locally advanced breast cancer, axillary lymph node dissection remains a pivotal component of surgical therapy. Apart from this, it has been mostly replaced by sentinel node biopsy. Complications after axillary dissection include wound infection, neuropathy, lymphedema and—most frequently—seroma. In this retrospective multi-centre study, we compared the use of LigaSure^TM^ with monopolar electrocautery regarding perioperative outcome.

**Methods:**

A retrospective data analysis from female breast cancer patients who underwent axillary dissection at two breast centres in Austria that are using two different surgical techniques was performed for this study. We compared the rate of complications and re-operations, length of hospital stay, time to drain removal, total drain fluid, seroma formation after drain removal, number of seroma aspirations and total seroma fluid.

**Results:**

Seventy one female patients with a median age of 63 (30–83) were included in this study. In 35 patients LigaSure^TM^ and in 36 monopolar cautery was used for axillary dissection. There was no significant difference regarding intraoperative complications and rate of re-operations between the two groups (2.9 vs. 5.6%; p = 1 and 2.9 vs. 13.9%; p = 0.199). The time to drain removal and the length of hospital stay was similar in both groups. A significant difference in the occurence of postoperative wound infection could also not be shown. However, we found a significantly smaller total drain fluid in the LigaSure^TM^-group compared to the cautery-group (364.6 ml vs. 643.4 ml; p = 0.004). Seroma formation after drain removal was more frequent in the LigaSure^TM^-group (68.6 vs. 41.7%; p = 0.032) with a higher number of outpatient seroma aspirations (2.0 vs. 0.9; p = 0.005).

**Conclusion:**

LigaSure^TM^ and monopolar cautery provide equivalent techniques in axillary lymph node dissection with comparable postoperative outcomes.

## Background

Surgical therapy in breast cancer which is the most frequently diagnosed cancer in women worldwide [1] has progressed over the centuries and became less radical and more focused on skin preserving and tissue sparing procedures. Likewise, the surgical approach to the axilla underwent significant changes in the last decades [2]. Axillary lymph node dissection (ALND) used to be the gold standard to control local recurrence and to increase overall survival (OS) and was therefore conducted systematically. This approach also allowed axillary lymph node staging in patients with invasive breast cancer. Sentinel lymph node biopsy (SLNB) which was first described by Gould et al. in 1960 [3] broke the dawn of a new era and mostly replaced conventional ALND as a standard procedure especially in early breast cancer. Multiple randomized clinical trials demonstrated that SLNB is a valid method for axillary lymph node staging as it reflects the overall axillary lymph node status with no significant differences in disease free survival and overall survival in comparison to ALND [4, 5]. For patients with breast cancer, SLNB is associated with fewer side effects when compared to ALND including postoeprative lymphedema and as a consequence offers better arm mobility and overall an improved quality of live [6, 7]. However, selected patients with locally advanced cancer with clinically positive axillary lymph nodes or macrometastasis in SLNB may still have an indication for ALND. According to current breast cancer treatment guidelines patients with histological pT1-pT1/cN0-cancers and one or two positive sentinel lymph nodes, who underwent breast conserving surgery (BCS) and subsequently irradiation, should not be treated with axillary dissection [8]. After mastectomy or BCS without postoperative irradiation adjuvant axillary dissection or axillary irradiation should be performed [8, 9]. In cases with exclusive micrometastasistargeted axillary therapies including axillary dissection or irradiation is not indicated [10, 11]. Patients after primary systemic therapy with pretherapeutic positive lymph node status in biopsy (cN1) and negative or positive lymph node status after systemic therapy (ycN0) should undergo axillary dissection [12, 13]. The most frequent surgical complication after axillary dissection is seroma formation with reported incidences between 18 and 74% [14–19]. Further possible complications associated with ALND include hematoma, pain, cutaneous necrosis, wound infection and sequela like weakness of the upper arm, limited motion of the shoulder with or without stiffness, reduced grip strength, paresthesia and lymphedema [20–23]. Most approaches with the goal to reduce the risk of seroma formation including physiotherapy, external compression or the intraoperative use of hemostatic adhesives have failed [24, 25]. However, the standardized use of closed suction drains in wound management could decrease the incidence and degree of postoperative seroma formation following ALND significantly [19]. On the other hand, drainages may cause pain, discomfort, restrict the mobility of the arm and increase the risk of secondary seroma infection, especially in cases with prolonged drain insertion [16, 25, 26]. Examined predictive factors for postoperative seroma formation are patient´s age, breast size and the number of involved nodes [27]. Another considerable factor that influences the occurrence of postoperative seroma is supposed to be surgical technique. Besides monopolar and bipolar cautery, scissors and cold knife, new devices like LigaSure™ (LS) (Covidien, CO, USA) promise a reduction of postoperative complications due to a superior vessel sealing system while inducing lower cellular damage. LS allows the sealing of vessels with diameters up to 7 mm. It´s safety, efficacy and time-saving use has already been examined in different surgical fields including colorectal surgery and thyroid surgery [28–33]. Regarding hemostasis, suture ligation or the use of clips for vascular blood vessel control during surgical dissection were shown to be associated with prolonged operative times and complications partially due to the sequalae of dislocations of tied knots and clips when compared to surgical dissection using LS for hemostasis [34]. Electrocautery (EC) on the other hand produces thermal damage to the surrounding tissue and thereby increases the risk of seroma formation [35]. However, the actual benefits of LS compared to EC in terms of postoperative seroma, drainage and hospital stay still remain unclear.

Thus, the aim of this study presented is the comparison of LigaSure™ versus EC combined with vascular ligation and vascular clip used for hemostasis control during axillary dissection in women suffering from breast cancer. Investigated study endpoints were intraoperative functional aspects, immediate postoperative outcomes and postoperative quality of life.

## Methods

### Study design

This retrospective study was conducted at two breast cancer centers in Graz, Austria. Female patients with the diagnosis breast cancer who underwent ALND for breast cancer treatment between 2013 and 2019 at the Department of General Surgery, University Hospital Graz and the Department of Surgery, Landeskrankenhaus Graz II were included in this study. The study protocol has been approved by the local Ethics Committee prior to data collection.

### Patients

Only female patients diagnosed with invasive breast cancer that underwent ALND were included in the study. All patients treated at the University hospital Graz were operated using EC and formed the EC group. The LS group included all patients from the Landeskrankenhaus Graz II, who were all operated using LigaSure™.

### Surgical technique

Patient preparation, incision and general surgical approach did not differ between the two centers. All patients were placed with the arm positioned 90° aside. The incision was performed longitudinal. The whole dissection was performed solely using either EC combined with vascular ligation and vascular clip or LS. In all patients, level I–II lymph nodes were resected while preserving the long thoracic and the thoracodorsal neurovascular bundles at all times. A preservation of all sensoric nerves like the second inercostal brachial nerve could not be achieved in every case. A closed suction drain was placed in the axillary fossa in both groups.

### Data collection

Data collection was carried out retrospectively in an anonymized form. Patient's data were obtained from the statewide connected digital clinical database “Medocs”. The following data was obtained: Patient´s characteristics—age, body mass index (BMI), physical status classification system of the American Society of Anesthesiologists (ASA), performance status of the Eastern Cooperative Oncology Group (ECOG), menopause status, familial disposal, therapy regimen (preoperative chemotherapy), operative parameters like intraoperative complications, rate of operative revisions, postoperative parameters including time to drain removal, totally drained fluid in ml, hospital stay in days, seroma after drain removal (yes/no), number of outpatient seroma aspirations, total seroma volume in ml, postoperative wound infections (yes/no), the histological type of the carcinoma, and the number of removed and involved lymph nodes.

### Statistical analysis

Statistical evaluation was performed using the software program SPSS 25 (Statistical Package for Social Sciences, Inc.; Chicago, IL, USA). For the comparison of both groups Wilcoxon–Mann–Whitney tests and Fisher´s exact tests were used to compare continuous data and binary data, respectively. Univariate and multivariate regression analysis was used to evaluate the impact of the surgical method for blood vessel control during dissection on surgical and immediate postoperative outcome. For multivariate regression, the following parameters were included in the analysis: Age at time of surgery, ASA, ECOG performance status, preoperative chemotherapy, surgical technique. The significance level was defined with *p-values* < 0.050.

## Results

### Patient's characteristics

Thirty six patients were included in the EC group and 35 in the LS group. Median age of all investigated patients was 63.4 (30–83). No significant differences could be detected between both study groups for age, BMI, ASA, ECOG performance status, menopause status or familial disposition for breast cancer. An overview of patient´s characteristics is given in Table 1.

**Table 1:** Patient's characteristics

Technique	Age	BMI	ASA	ECOG	Preoperative CTX (%)
LS	64.5 ± 13	27 ± 5.5	39.3 ± 1.1	0.3 ± 0.4	42.9
n	35	35	35	35	35
EC	62.3.3 ± 12.2	28.1 ± 5.9	39 ± 1.1	0.2 ± 0.4	50
n	36	36	36	35	34

Age (years), BMI (kg/m^2^), ASA and ECOG at the time of examination given in mean ± SD; number of patients after preoperative chemotherapy (CTX) given as a percentage

### Operative details

The rate of intraoperative complications and operative revisions did not differ significantly between the two groups. Intraoperative complications occurred in 5.6% of the EC and 2.9% of the LS group (*p* = 1.000). 13.9% of the women operated using EC and 2.9% of those operated with LS had to be re-operated due to postoperative complications—mostly due to bleeding—or due to R1-resection (*p* = 0.199). We also compared the mean duration of the intraoperatively placed closed suction drainage and found no significant difference between the EC and the LS group (5.3 ± 2 vs. 5.2 ± 2.4 days; *p* = 0.709). However, a significantly lesser amount of drained volume could be determined in the LS group (643.4 ± 500 vs. 364.6 ± 304.6; *p* = 0.004). Also, a significant impact of the surgical technique on the drained fluid could be shown in univariate (*p* = 0.006) and multivariate regression analysis (*p* = 0.012).

**Fig. 1:**
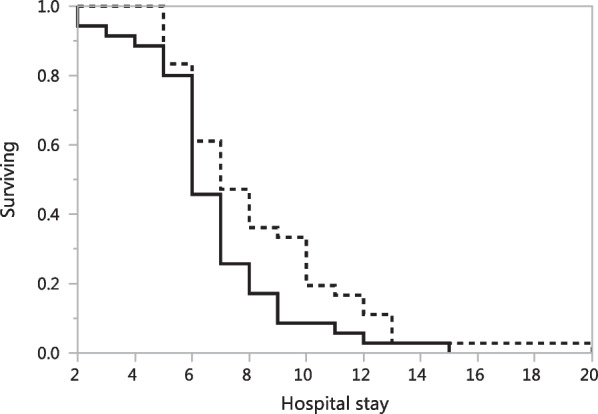
Shown is the Kaplan-Meier analysis of the influence of bleeding control with LigaSure™ (straight line) versus electrocautery (dotted line) on the length of hospital stay (days) demonstrating that those patients treated with LigaSure™ had significantly shorter hospital stays (p = 0.043, log rank test)

Kaplan-Meier analysis demonstrated that bleeding control with LS versus EC was associated with a significantly shorter hospital stay (p = 0.043, log rank test) (Fig. 1). Furthermore, multivariate Cox regression analysis revealed that the deployment of LS versus EC had a statically significant influence on shorter hospital stays (*p* = 0.041, hazard ratio (HR) = 1.692, 95% confidence interval of the HR: 1.024–2.823) independent of the age at surgery (years) (p = 0.155), ASA score (p = 315), ECOG performance status (p = 0.566), and preoperative chemotherapy (p = 0.158) which had no significant influence on the duration of the hospital stay.

**Fig. 2:**
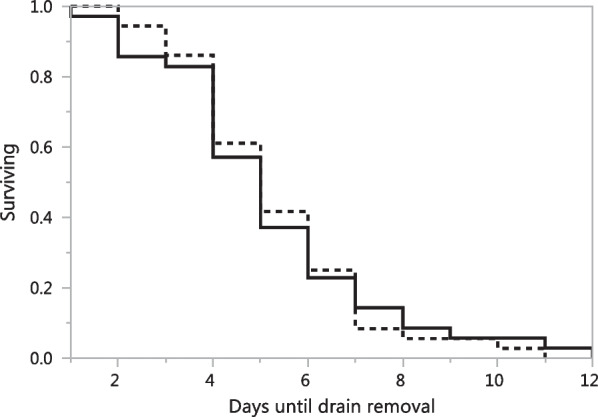
Shown is the Kaplan-Meier analysis of the influence of bleeding control with LigaSure™ (straight line) versus electrocautery (dotted line) on the number of days until drain removal demonstrating no significant difference (p = 0.932, log rank test)

Kaplan-Meier analysis demonstrated that bleeding control with LS versus EC had no statistically significant influence on the numbers of days until drain removal (p = 0.932, log rank test) (Fig. 2). Furthermore, multivariate Cox regression analysis revealed the deployment of LS versus EC surgical technique had no statically significant influence on the number of days until drain removal (p = 0.981).

What we did find was a significantly lower percentage of patients suffering from postoperative seroma due to axillary seroma which needed outpatient treatment after drain removal in the EC group (41.7%) when compared to the LS group (68.6%) (*p* = 0.032). Regression analysis confirmed this assumed influence of the surgical device on seroma formation (*p* = 0.025 for both uni- and multivariate analysis). Consequently, a significantly higher number of outpatient seroma aspirations was performed in the LS group (2 ± 2.1 vs. 0.9 ± 1.5; *p* = 0.005). However, the total seroma volume was higher in the EC group although this difference did not reach statistical significance (0.9 ± 1.5 vs. 2 ± 2.1; *p* = 0.005). Postoperative wound infections occurred in 8.3% of women in the EC group and 11.4% of those in the LS group (*p* = 0.710). The number of resected lymph nodes was similar in the two groups (14.7 ± 5.3 and 17.5 ± 7.4, respectively; *p* = 1.141, Wilcoxon–Mann–Whitney-Test).

## Discussion

This study focuses in contrast to previous trials specifically on the comparison of LS vs. EC.

New surgical devices promise a dramatic change regarding intra- and postoperative complications in breast surgery. Several randomized trials demonstrated a reduction of operative time, intraoperative blood loss and postoperative pain by the use of LS [36].

Axillary lymph node dissection still remains an essential part of modern breast cancer therapy although it´s usage has been restricted lately. Among possible complications following ALND, seroma formation is known to be the most common, but can be avoided be the usage of closed suction drains. Compared to the conventional harmonic scalpel, LS seems not only to reduce postoperative seroma formation and lymphedema, but also operative time, perioperative complications—especially blood loss and total volume and duration of drainage [34].

Our study compares two of the most commonly applied surgical devices in breast surgery –monopolar EC and LS.

We expected a reduction of total volume and duration of drainage in the LS group due to the already proven adequate sealing of lymphatics by electrothermal sealing and pressure [37]. Thus, Magri et al. found in a retrospective study including 187 patients a shorter duration of drainage after ALND using LS compared to vascular clips [38].

While our data shows that the totally drained volume during hospital stay was significantly reduced in the LS group, after release from hospital, patients in the LS group developed significantly more frequently seroma. Considering these two observations, the higher number of seroma formation in the LS group could be seen as a result of early drain removal which was also shown in our analysis, although without reaching statistical significance.

In our hospital, suction drainage is removed as of the second postoperative day if the drained fluid undershoots 50 ml and there is no sign of wound infection. However, sometimes patient-related factors like pain that is caused by the drainage may play a role and make the decision to remove the drainage an individual one.

Similar to our results, Tukenmez et al. showed in a prospective study (n = 33) smaller drain volumes and shorter durations of drainage after ALND using LS when compared to EC and suture ligation [39].

In our population, postoperative seroma formation was more frequent in the LS group, which is in line with significantly more frequent outpatient seroma aspirations after surgery with LS. Interestingly, the volume of aspirated seroma was lower in the LS group without reaching statistical significance. We believe that this observation is likely a result of user-dependent differences in aspiration handling. The increased number of seroma aspirations in the LS group was not associated with increased postoperative wound infections. Moreover, other authors also did not find a significant difference in postoperative wound infection between patients that underwent ALND using LS instead of conventional devices [35, 40]. In contrast to these observations, Gunn et al. could show a higher number of symptomatic axillary seroma in patients who developed postoperative wound infections [41].

Cox regression analysis demonstrated a significantly shorter hospital stay of patients operated with LS which potentially implies significant treatment cost advantages warranting further economic analysis. This finding correlates with shorter drainage durations and smaller amounts of totally drained fluids in the LS group.

In contrast to our results, Antonio et al. found in a prospective randomized trial including 100 women with breast cancer who underwent ALND no improvement by using LS instead of conventional dissection in terms of duration of drainage and amount of drained fluid [42]. In accordance with our findings, the incidence of seroma after drain removal was significantly higher in the LS group [42]. On the other hand, Cortadellas et al. reported a reduction of postoperative seroma punctures and total amount of aspirated fluid in their prospective randomized study (n = 100) when applying LS compared to conventional ALND using EC, suture ligation or vascular clips [40]. They furthermore determined an association of LS with reduced intraoperative blood loss, shorter duration of axillary surgery and—similar to our findings—fewer days of suction drain and shorter lengths of hospital stay.

A clear advantage of LS concerning intraoperative complications and need for re-operation, respectively, could not be demonstrated in our study. Neither did the number of retrieved lymph nodes differ significantly in our population. This finding has already been reported by several authors who compared LS with conventional surgical techniques in ALND [35, 40, 42].

The presented study has some limitations, including the retrospective study design, which opens the door for selection bias and also measurements bias. Vulnerability to bias also results from the reservation of one surgical device at each centre.

## Conclusion

The results presented here approve LS to be a valuable and partially superior alternative to conventional axillary lymph node dissection using electrocautery, although clear advantages could only be proven in certain outcome parameters including shorter lengths of hospital stay.

## Data Availability

The datasets used and/or analysed during the current study are not publicly available due to the local data protection law but are available from the corresponding author on reasonable request.

## Abbreviations

ALND: Axillary lymph node dissection
OS: Overall survival
SLNB: Sentinel lymph node biopsy
BCS: Breast conserving surgery
LS: LigaSure™
EC: Electrocautery
BMI: Body mass index
ASA: American Society of Anesthesiologists
ECOG: Eastern Cooperative Oncology Group
